# Telehealth Pilot Study of the Effects of a Naturalistic Developmental Behavioral Intervention on Child Social Communication Outcomes in a Community Mental Health System

**DOI:** 10.3390/bs15091171

**Published:** 2025-08-28

**Authors:** Jessie Greatorex, Diondra Straiton-Webster, Brooke Ingersoll

**Affiliations:** 1 Department of Psychology, Michigan State University, East Lansing, MI 48824, USA; diondra.straiton@pennmedicine.upenn.edu (D.S.-W.); ingers19@msu.edu (B.I.); 2 Department of Psychiatry, Perelman School of Medicine, University of Pennsylvania, Philadelphia, PA 19104, USA; 3 Leonard Davis Institute of Health Economics, University of Pennsylvania, Philadelphia, PA 19104, USA

**Keywords:** NDBI, autism, caregiver-mediated intervention, Medicaid, community mental health, project ImPACT, telehealth

## Abstract

Little is known about the effectiveness of caregiver-mediated naturalistic developmental behavioral interventions (NDBIs) implemented via telehealth for autistic children served in under-resourced settings. This mixed methods pilot study examined social communication outcomes for autistic children whose families received a caregiver-mediated NDBI in a community mental health setting. Twenty-one families of Medicaid-enrolled autistic children aged 2–6 received Project ImPACT (a caregiver-mediated NDBI) via telehealth. Caregivers completed the Autism Impact Measure at 5 timepoints. We fit three, 2-level multilevel models to estimate the effect of time (weeks of Project ImPACT), child age (mean-centered), and the interaction of time x age on the Autism Impact Measure domains of Communication, Social Reciprocity, and Peer Interaction. Six caregivers completed follow-up interviews, which were analyzed using the framework method. There were statistically significant decreases in caregiver-reported peer interaction challenges. Decreases in communication challenges approached statistical significance. Scores for social reciprocity challenges did not significantly change over time. Six qualitative themes centered around how the child- and family-centered aspects of the NDBI strategies led to improvements in the children’s social communication outcomes and suggestions for improving Project ImPACT. Preliminary findings suggest that NDBIs may be feasible and potentially effective in under-resourced settings.

## 1. Introduction

Naturalistic Developmental Behavioral Interventions (NDBIs) are an emerging class of interventions that integrate principles of Applied Behavior Analysis (ABA) with developmental science to support social communication development in young children (Schreibman et al., 2015). There is a growing evidence base for the effectiveness of NDBIs in the treatment of young autistic children and those at high likelihood of autism, with several recent meta-analyses indicating significant improvements in developmental outcomes (Crank et al., 2021; Sandbank et al., 2023; Tiede & Walton, 2019).

In caregiver-mediated NDBIs (CM-NDBIs), a clinician teaches one or more caregivers to implement intervention strategies with their child to support their development, particularly in the area of social communication. A number of studies have shown positive effects of CM-NDBIs on child and family outcomes (Jurek et al., 2023; Pacia et al., 2022; van Noorden et al., 2024) and CM-NDBIs are now considered best practice in the treatment of young children with or at high likelihood of autism (Zwaigenbaum et al., 2015). However, the existing evidence base for CM-NDBIs lacks sufficient representation of under-resourced and minoritized children (Rogers et al., 2022; van Noorden et al., 2024), and several studies have reported lower engagement among under-resourced families (Carr et al., 2016; Carr & Lord, 2016; Kasari et al., 2014). These families are often multiply marginalized by systems of oppression (e.g., classism, poverty, racism), limiting access to, and the uptake of, specialized autism services like CM-NDBIs (Smith et al., 2020). Caregiver-mediated interventions have the potential to increase the dosage of intervention strategies for a child because family members can implement the strategies even when the provider is not present (Rocha et al., 2007). This may be particularly impactful for families from low-income backgrounds, who receive fewer hours of early intervention services than families from higher-income backgrounds (Aranbarri et al., 2021).

This discrepancy underscores the necessity for further research on the effectiveness of CM-NDBIs in diverse community settings across the United States. The social validity of behavioral interventions for autism may vary by cultural group, and cultural norms and social expectations influence the appropriateness of techniques like prompts and positive reinforcement. Few studies have specifically investigated the social validity of behavioral autism interventions in families from non-Western cultures, though most have demonstrated mostly positive attitudes towards these interventions (e.g., Sivaraman & Fahmie, 2020; Larson et al., 2020).

Current evidence points to low utilization rates of CM-NDBIs in community settings, particularly within systems serving families from low-income backgrounds (Straiton et al., 2021a; Tomczuk et al., 2022). While research within the Part C system has demonstrated that early intervention providers can be trained to implement CM-NDBIs, there is limited information on the effects on child outcomes (Rogers et al., 2022). Providers report significant barriers to using CM-NDBIs with low-income and minoritized families, such as logistical challenges with transportation and scheduling, and difficulties with engaging caregivers in services (Straiton et al., 2021b; Tomczuk et al., 2022). Though these challenges have been well-documented, few studies have focused on the effects of CM-NDBIs on child and family outcomes for families of minoritized autistic children, with all studies including a small sample of less than 35 families. One study of Spanish-speaking families predominantly from lower-income backgrounds in the United States demonstrated increased social communication skills (Pickard et al., 2024). Another found improvements in social communication outcomes for young autistic children in South Africa (Rieder et al., 2023). Nevertheless, other studies of caregiver-mediated NDBIs with marginalized families in under-resourced service systems have not found statistically significant improvements in child outcomes (Pellecchia et al., 2024; Stahmer et al., 2020).

Telehealth delivery of caregiver-mediated interventions can address many barriers faced by minoritized families, especially those living in service access deserts and those with limited financial and transportation resources (Straiton et al., 2021a, 2021b; Alatar et al., 2023). Few studies have examined child outcomes for CM-NDBIs delivered via telehealth. A recent review of nine studies of telehealth-delivered CM-NDBIs demonstrated mixed and mostly null results for child outcomes across studies, with the authors concluding that there is limited evidence for improvements in child social communication outcomes (Alatar et al., 2023). However, only two of these studies enrolled families from predominantly low-income backgrounds. Moreover, to our knowledge, only one study to date has investigated the use of CM-NDBIs via telehealth when delivered by *community providers* rather than staff at university-affiliated clinics (Simcoe et al., 2025), and no studies have investigated telehealth-delivered CM-NDBIs with community providers at ABA clinics or in the community mental health context.

In Michigan, families from low-income backgrounds can access ABA services through the Medicaid Autism Benefit for youth under the age of 21. Our previous research identified very low utilization of caregiver training within this benefit, despite incentivizing ABA providers via higher reimbursement rates and caregiver-reported interest in such services (Casagrande, 2021; Straiton et al., 2021a). Further, use of telehealth to deliver caregiver training prior to the COVID-19 pandemic was extremely low (Straiton et al., 2021a), even though ABA providers identified telehealth as a facilitator that could significantly help mitigate logistical barriers (Straiton et al., 2021b). The current pilot study took place during the COVID-19 pandemic (between February 2021 and September 2022), which fueled an increased demand for telehealth service models. Our community partners indicated a desire to increase ABA providers’ use of telehealth to deliver caregiver-mediated interventions, and particularly caregiver-mediated NDBIs, within the Medicaid Autism Benefit.

Project ImPACT, a caregiver-mediated NDBI was adapted for use within the Medicaid system and can be effectively delivered via telehealth (Ingersoll et al., 2024). Building on our previous work, this mixed methods pilot study aimed to examine the preliminary effects of Project ImPACT on child outcomes when implemented over telehealth by community ABA providers within the community mental health system in Michigan. Our aims were as follows:

1. To what extent does Project ImPACT improve caregiver-reported social communication outcomes for autistic children receiving the intervention from novice clinicians in the community mental health system?
2. What are caregiver perspectives on the Project ImPACT strategies and their experiences with the intervention in this publicly funded service system?

## 2. Materials and Methods

### 2.1. Patient and Public Involvement

This study was developed in collaboration with community mental health agency leaders as part of a larger community-partnered project examining the potential active ingredients of a consultation model designed to improve implementation outcomes within the community mental health system. Caregivers and autistic people were not directly involved in study design, though caregivers provided feedback about the intervention during follow-up interviews.

### 2.2. Design

The present analysis of child and family outcomes is a secondary analysis of a larger pilot project. The primary outcomes were to examine the effect of consultation training activities on clinician fidelity, comparing fidelity scores at baseline to scores across three training phases (Straiton-Webster et al., in prep). Clinicians provided Project ImPACT to families of Medicaid-enrolled clients served in a community mental health system. This secondary analysis evaluated child and family outcomes of the intervention. Caregivers reported on child skills in questionnaires collected throughout the study period. Caregivers also completed exit interviews.

We used a QUAN → QUAL mixed methods approach (Palinkas et al., 2011), analyzing quantitative outcomes from the Autism Impact Measure (AIM; Kanne et al., 2014) before analyzing qualitative interview data. We provided equal emphasis to each source of data.

### 2.3. Changes to the Protocol

No changes to the study protocol were made after commencement.

### 2.4. Setting

Data were collected within the Michigan Medicaid Autism Benefit service system. In this system, state Medicaid funds are provided for applied behavior analysis (ABA) services for low-income youth aged 0–21 with a diagnosis of autism spectrum disorder.

### 2.5. Eligibility Criteria

Provider eligibility included being over 18 years of age, being qualified to bill through the Medicaid Autism Benefit, speaking English, and having at least one caregiver on their caseload who would be appropriate for Project ImPACT and who agreed to enroll in the study. Family eligibility included having a caregiver who: was the parent or legal guardian of an autistic child between the ages of 24 and 72 months who was enrolled in the Medicaid Autism Benefit, spoke English, and whose enrolled child had a community diagnosis of Autism Spectrum Disorder (ASD) established using the ADOS-2 (this was a requirement to receive services in this system). We did not assess for cognitive level or severity of autism features due to resource constraints of this pilot study. Demographics are reported in Table 1.

### 2.6. Intervention

Project ImPACT is a caregiver-mediated NDBI that can be delivered via telehealth using a parent coaching approach. Clinicians first completed the 6 h self-directed Project ImPACT Beginner e-Course over a two-week period. They then entered a baseline phase (3–9 weeks; randomized by agency) during which they followed the Project ImPACT coaching manual without consultation support, meeting weekly with enrolled families. Following baseline, clinicians received 12 weekly 1.5 h virtual group consultation sessions on Zoom with a Project ImPACT consultant (second author), a multiracial (Dominican, Irish, German) neurotypical, cisgender woman pursuing a PhD in clinical psychology with prior experience training over 50 clinicians in Project ImPACT.

Clinicians were instructed to deliver the Project ImPACT program once per week via a telehealth platform supported by their agency (e.g., Zoom, HIPAA-compliant Google Meet), for a total of 24 sessions, with a session length of 60 min per session. Sessions targeted social communication development. Caregiver sessions in Project ImPACT follow a consistent structure: (1) a review of between-session practice, (2) didactic teaching of a new NDBI strategy, (3) active caregiver practice with in vivo feedback, and (4) creation of a homework plan for continued practice.

### 2.7. Outcomes

Quantitative social communication outcomes were measured using the Autism Impact Measure (AIM), a caregiver-report questionnaire assessing child skills and autism-related challenges (Kanne et al., 2014). The AIM has strong psychometric properties and is sensitive to change in response to Project ImPACT (Mazurek et al., 2020). The AIM assesses symptoms across 5 domains: Repetitive Behavior, Atypical Behavior, Communication, Social Reciprocity, and Peer Interaction (Mazurek et al., 2020). Given the social communication focus of Project ImPACT, we examined the Communication, Social Reciprocity, and Peer Interaction subscales. Caregivers completed the AIM online at five timepoints: intake (baseline), during the intervention (4, 8, and 12 weeks post-intake), and follow-up (8 weeks post-intervention).

For qualitative results, semi-structured interviews explored caregiver perceptions of changes in child social communication outcomes and experiences with the intervention. Caregivers were invited to participate in a semi-structured Zoom interview with a study staff member not involved in the Project ImPACT training. The interviewer was a neurotypical, cisgender, white woman from an upper-middle-class background, pursuing a bachelor’s degree in psychology, with experience working as a behavior technician and caregiver for autistic people. Interviews lasted approximately 30 min, were audio-recorded, transcribed automatically by Zoom, and manually corrected by the interviewer.

### 2.8. Sampling and Sample Size

Of the 21 caregivers enrolled in the study, 17 completed assessments and 6 completed follow-up interviews. The sample size was determined by project score and resource constraints and was thus not powered for statistical significance testing.

### 2.9. Randomization

Participants were not randomized because all children received the intervention (Project ImPACT). Caregivers reported on baseline child skills at intake and throughout the intervention period. We used 2-level multilevel models to examine changes in outcomes over time, relative to mean baseline scores.

### 2.10. Statistical Methods

For quantitative analyses, a series of multilevel models were fit using restricted maximum likelihood estimation to estimate the extent to which Project ImPACT was associated with changes in caregiver-reported child social communication outcomes over time. Observations (i.e., caregiver ratings on the AIM) were nested within each child. Fixed effects included time (i.e., weeks of Project ImPACT), child age at intake, and the interaction of child age x time. We included the interaction of age and time because previous work has demonstrated significant interactions between age and time spent receiving intervention, with a lower magnitude of intervention effects for older children (Peterson et al., 2024). Because higher scores on the Autism Impact Measure indicate a higher number of challenges, negative values in the fixed effects represent a decrease in autism-related challenges. Models also included a random component for the intercept. This random component allowed the model to vary for each child’s baseline score (i.e., intercept). We hypothesized that weeks of Project ImPACT would be associated with decreases in social communication challenges, with stronger associations for younger children.

For qualitative analyses, we used inductive coding and framework analysis (Ritchie & Spencer, 1994) to analyze the caregiver interviews. Coders also reflected on their social identities and potential biases throughout the coding process (i.e., reflexivity). The first author read through all transcripts and created notes about initial impressions of the data. Thirteen inductive codes were developed following a close reading of all of the transcripts. Once the codebook was finalized, the first author then read each transcript and applied codes line by line; the second author then audited the first author’s coding, noting any questions and disagreements about the codes that were applied (Saldaña, 2015). The second coder was able to view the first author’s coding and memos while auditing. The first and second authors reviewed the entirety of the dataset, resolving any disagreements by consensus.

## 3. Results

### 3.1. Participant Flow and Flow Diagram

**Figure 1:**
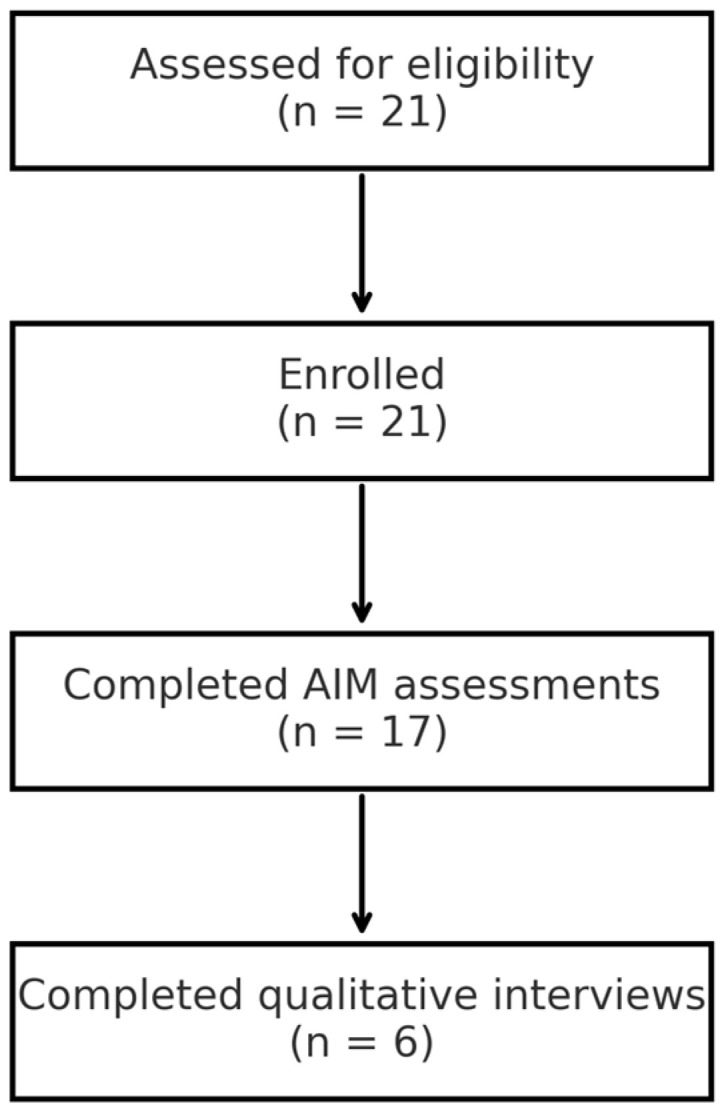
Participant flow diagram.

Of 21 eligible caregiver–child dyads approached, all enrolled in the study. Seventeen children from five agencies provided complete AIM data. Six caregivers participated in follow-up interviews. See Figure 1 for a flow diagram to depict recruitment and retention.

### 3.2. Recruitment

Recruitment occurred during the COVID-19 pandemic, between February 2021 and September 2022. Agency leaders connected the study team with eligible clinicians at their clinic who indicated interest in the research project. Enrolled clinicians facilitated family recruitment by connecting the study team with eligible caregivers on their caseload.

### 3.3. Intervention Delivery

Clinicians were asked to deliver the intervention sessions once per week for 60 min via telehealth. Overall, clinician fidelity in this sample was well below the 80% fidelity benchmark for Project ImPACT coaching fidelity, with the mean fidelity score being 63%.

### 3.4. Baseline Data (Demographics)

Clinicians were predominantly white and non-Hispanic (100%), women (90%), with a mean age of 32 years (range 23–58 years). Most were Board-Certified Behavior Analysts (95%). Children were mostly boys (59%), white (73%), and non-Hispanic (82%), with a mean age of 55 months (range 24–76 months). Mean household income was $36,299 per year, with an average of four people per household. See Table 1 for full demographics.

### 3.5. Quantitative Results

Models included 62 questionnaires across 17 children from 5 agencies. 

#### 3.5.1. Peer Interaction

At intake, the average Peer Interaction score was estimated at 22.53 (95% CI: 19.89 to 25.16). The effect of Time was negative and statistically significant (β = −0.25; 95% CI: −0.45 to −0.04; *p* = 0.021), indicating that peer interaction challenges declined over time. In contrast, neither Child Age (β = −0.18; 95% CI: −0.39 to 0.03; *p* = 0.119), nor the interaction between Time and Child Age (β = 0.01; 95% CI: −0.007 to 0.029; *p* = 0.244), was significantly associated with trajectories of peer interaction challenges.

**Figure 2:**
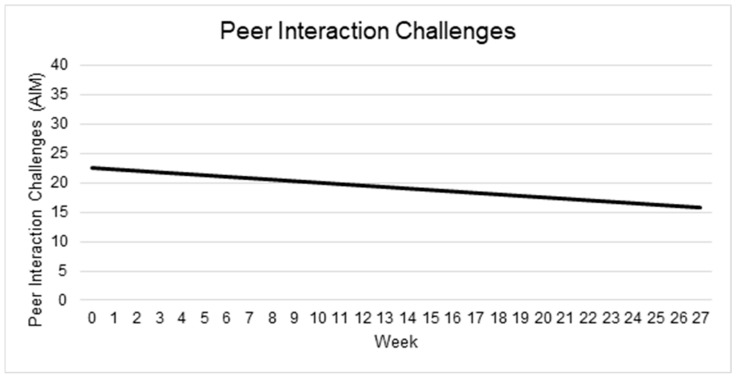
Changes in peer interaction challenges over time. Note: Higher scores indicate more challenges.

Between-child variability in baseline Peer Interaction scores was moderate, with a random-intercept standard deviation of 4.81 (95% CI: 3.06–6.85), while the within-child (residual) standard deviation was 3.46 (95% CI: 2.79–4.21). The resulting intraclass correlation coefficient was approximately 0.66, indicating that about two-thirds of the total variability in Peer Interaction scores lay between children rather than over time within children. See Table 2 and Figure 2. 

#### 3.5.2. Communication

At intake, the average Communication score was estimated at 40.09 (95% CI: 34.84 to 45.32). The effect of Time on Communication was negative and approached statistical significance (β = −0.27; 95% CI: −0.56 to 0.04; *p* = 0.092), indicating no reliable time trend, though the trend was in the expected direction. Similarly, neither Child Age (β = −0.21; 95% CI: −0.63 to 0.20; *p* = 0.335) nor the interaction between Time and Child Age (β = −0.003; 95% CI: −0.030 to 0.024; *p* = 0.839) were significantly associated with Communication scores.

**Figure 3:**
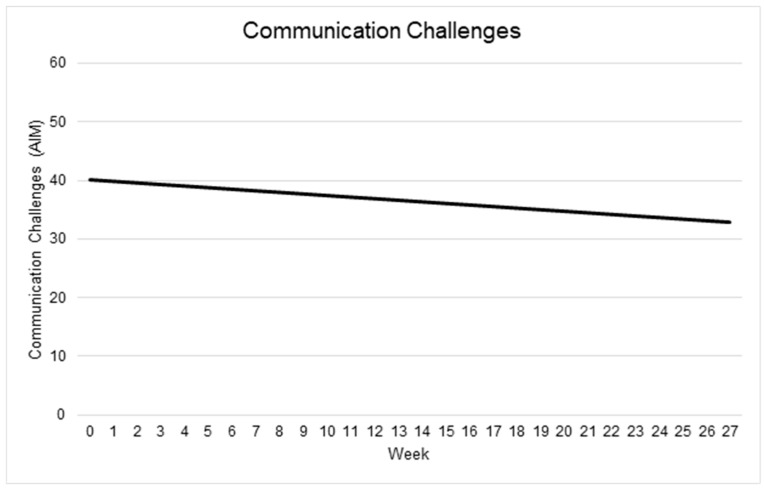
Changes in communication challenges over time Note: Higher scores indicate more challenges.

Between-child variability in baseline Communication score was substantial (SD = 10.25; 95% CI: 6.82–14.32), while within-child (residual) SD was 5.13 (95% CI: 4.14–6.26). The intraclass correlation indicated that roughly 80% of the variability lay between children, rather than over time within children. See Table 3 and Figure 3.

#### 3.5.3. Social Reciprocity

At intake, the mean Social Reciprocity score was 27.30 (95% CI: 24.58–30.01). Over time, there was a non-significant decrease of 0.14 points per week (95% CI: −0.37 to 0.10; *p* = 0.265). Neither Child Age (β = −0.09; 95% CI: −0.31 to 0.13; *p* = 0.431), nor its interaction with Time (β = 0.011; 95% CI: −0.0097 to 0.032; p = 0.309) were significantly associated with Social Reciprocity scores.

**Figure 4:**
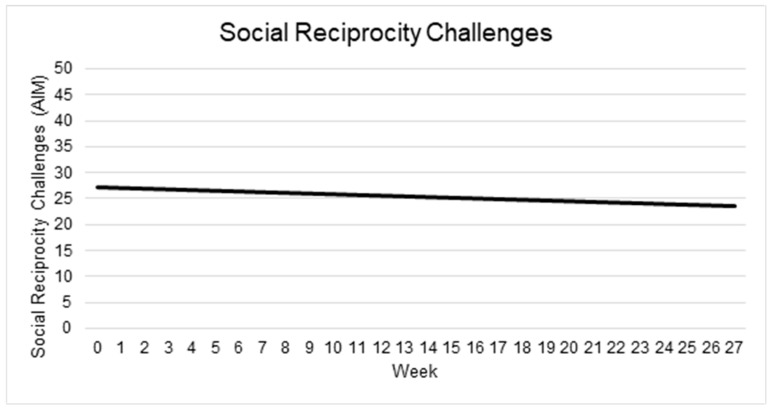
Changes in social reciprocity challenges over time. Note: Higher scores indicate more challenges.

Substantial baseline variability existed between children (SD = 4.70; 95% CI: 2.83–6.83), while within-child (residual) variation was comparable (SD = 4.06; 95% CI: 3.28–4.95). The intraclass correlation coefficient was 0.57, indicating that greater than half of the total variation in Social Reciprocity scores occurred between, rather than within, children. See Table 4 and Figure 4.

### 3.6. Qualitative Results

#### 3.6.1. Project ImPACT Changes How Caregivers Interact with Their Child

All caregivers reported a change in the way they spoke to, played with, and interacted with their child following participation in Project ImPACT. Examples of these changes include increasing the length of time for one-on-one play time with their child, incorporating new activities in their play, increased animation during caregiver–child interactions, having more patience, and following the child’s lead in play.

#### 3.6.2. Project ImPACT Teaches Caregivers to Use NDBI Strategies That Expand the Child’s Communication

Caregivers often reported on how their child’s communication skills grew as a direct result of their use of the Project ImPACT intervention strategies. For example, one caregiver reported that in the last six months, they had “seen so much growth in his communication, like, even if it’s not typical communication, he is signing more because I’m stopping and waiting for a response.” Caregivers specifically described the following Project ImPACT strategies as being effective at improving their child’s communication: communicative temptations (i.e., setting up an enticing situation and waiting with an anticipatory look so that the child is more likely to request an item/activity), prompts for communication (i.e., using communication prompts with natural reinforcement and increasing support if needed), and modeling and expanding communication (i.e., narrating what the child is seeing/doing using language that is slightly more complex than the child’s current level). For example, one caregiver mentioned the strategy of labeling the child’s play instead of asking questions to support the child’s use of communication.

#### 3.6.3. The NDBI Strategy of Following Your Child’s Lead Supported Child Social Engagement

Most caregivers reflected on the importance of letting their child take the lead in play, rather than trying to control the way that their child plays. This is a Project ImPACT intervention strategy called “Follow the child’s lead.” By allowing the child to guide the play, caregivers reported letting go of the pressure that they put onto themselves about constantly feeling they should be prompting their child. One parent expressed: “If she wants to spin around in a circle, I’ll spin around in a circle. If she wants to go to the park to do whatever, I go to the park. So, I just follow her lead, and I realized any type of play is play, and that maybe today you don’t have to learn a new skill, just maybe today is to just follow her lead and play.” Caregivers described that, when they used this strategy, they often found that the child was more engaged in the play and the interaction lasted longer: “I’m focusing on her, you know doing things she wants to do, and then the playtime can last a bit longer.”

#### 3.6.4. Project ImPACT Strategies Increased Children’s Social Engagement

Most caregivers reported an increase in child-initiated social engagement due to their participation in Project ImPACT. Increased social engagement was observed across multiple social contexts, including increased engagement with parents, siblings, classroom peers, and extended family members. For example, one caregiver reported that their child had begun “acknowledging people, even extended family that we only see a handful of times a year.” Caregivers reported that children began to show more of an interest in initiating social contact, even with others outside of their immediate family.

#### 3.6.5. Project ImPACT Increased Peer Interaction

Many caregivers specifically noted an increase in their child’s interaction with peers. One caregiver reflected on a particularly significant moment of growth, sharing: “By the end of Project ImPACT, he was requesting to play with [a peer]. We actually put, like, a picture of her on his iPad so he could ask to go to the room and play… You know, that was massive… recognizing someone wanted to play with him and reciprocating that back was huge.” Even though peer interaction was not a direct focus of the intervention, caregivers reported that the communication skills fostered through Project ImPACT enabled children to initiate and respond to their peers effectively.

#### 3.6.6. Project ImPACT Supported the Inclusion of Additional Family Members in the Child’s Intervention Services

Caregivers also reported that the strategies they learned through Project ImPACT could be taught easily to other family members, including other caregivers, grandparents, and siblings. One parent mentioned that: “Project ImPACT… was able to teach not just my child, but the adults in our life as well, you know, how to how to interact with him and how to kind of get through to him and get him to respond to us.” Family members who did not directly learn the intervention were also able to utilize the strategies, potentially providing increased opportunities for learning and generalization of skills across more contexts.

#### 3.6.7. Caregivers Noted Suggestions About Improving Project ImPACT Materials

When asked about aspects of Project ImPACT they did not find helpful or would like to see improved, half of the caregivers mentioned a lack of diversity in the video examples that were used to model NDBI strategies. Specifically, they noted that the examples did not always reflect the range of children’s communication abilities or developmental levels, making them less relatable. As one caregiver shared: “I kind of wish that they had more of the video examples, with more than one child and parent to show interactions because the spectrum is so wide that not all kids function at the same level.”

In addition, some caregivers highlighted challenges with telehealth, both in regard to practicing intervention strategies and receiving feedback via telehealth. While many acknowledged the convenience of remote sessions, some caregivers found it difficult to keep their child engaged while on screen. Others felt that the format reduced the personal connection with the clinician, particularly during moments that required interactive coaching: “It takes out some of the personal, the trying to actually like one-on-one work with somebody closer, especially when interacting with my child is part of the session.”

Despite these challenges, caregivers expressed appreciation for other aspects of the program. Many valued having a caregiver manual to refer back to throughout the week, which helped support independent practice. The consistency of weekly sessions was also seen as beneficial. Some caregivers suggested expanding the program to include follow-up sessions or extending its duration to better support long-term use of the strategies: “I wish it was a little longer, and I wish it was something that followed on with the kid’s development. I really thought it was helpful, so I was hoping it was something we could revisit in maybe six months or a year.”

See Table 5 for a joint display of the qualitative and quantitative data.

## 4. Discussion

### 4.1. Child Outcomes

This pilot study sought to understand the extent to which a caregiver-mediated NDBI, Project ImPACT, improved social communication outcomes in young autistic children served by novice clinicians in the community mental health system. It is important to note that this is a pilot study with a relatively small sample (N = 21) of families and results should be interpreted with caution.

To our knowledge, this is the first study to examine the effects of a CM-NDBI delivered by community clinicians via telehealth with autistic children from low-income backgrounds. Though our sample size was small (N = 17 families), results from our quantitative and qualitative datasets converged and suggest that caregivers of children who received Project ImPACT in this under-resourced setting noted improvements in some social communication outcomes for their children. Our pilot study demonstrated that our data collection procedures were feasible, with a representative sample of 21 families enrolled and complete questionnaire data from 81% of enrolled families. Using mixed methods allowed for a richer description of child outcomes than solely relying on underpowered quantitative analyses alone.

Our quantitative results indicated statistically significant improvements in peer interaction skills and marginally significant improvements in communication skills. These findings converge with findings from the qualitative interviews, in which caregivers described increases in peer interaction and increased complexity of communication skills (e.g., using more complex skills than skills at baseline, using words more frequently). Though this pilot study is underpowered, these results are encouraging. This study took place within an under-resourced community mental health system and was delivered by novice clinicians, many of whom were not implementing Project ImPACT at fidelity; indeed, the average manual adherence score for clinicians in the study was 63%, well below the fidelity threshold of 80% (Straiton-Webster et al., in prep). It is possible that high clinician fidelity (>80%) may not be necessary for improvement in some child outcome domains. More research is needed to understand how clinician fidelity relates to child improvement.

The finding regarding improvements in peer interaction was particularly surprising given that peer social skills are not a direct target of the Project ImPACT intervention. However, this increase in peer interaction can be viewed as a valuable secondary outcome. It is likely that the communication strategies taught in Project ImPACT, such as turn-taking, imitation, and initiating or responding to communication, help equip children with foundational social tools. These tools may also generalize to interactions with peers, thereby naturally fostering more social engagement in group settings.

Contrary to our hypothesis, our quantitative results did not indicate significant improvements in social reciprocity challenges over time. This may be due to the complex social-cognitive ability that social reciprocity skills involve (Leach & LaRocque, 2011). As Project ImPACT is a caregiver-mediated intervention, it is possible that the low level of clinician manual adherence among these novice clinicians made it difficult for caregivers to successfully implement NDBI strategies to address social reciprocity skills. At the same time, our qualitative findings identified a number of themes that are consistent with improvements in social engagement. Thus, it is possible that the social reciprocity items on the AIM do not fully capture improvements in social engagement skills that are observable to caregivers.

Families of autistic children served in community mental health settings often face more barriers to receiving evidence-based practices, such as financial strain (Straiton et al., 2023; Tomczuk et al., 2022). It is encouraging that this pilot study provides some preliminary evidence of some improvements in social communication skills, despite several barriers that affected service provision, including limited financial resources in the Medicaid system, the COVID-19 pandemic and associated telehealth use for intervention delivery, and low rates of Project ImPACT coaching fidelity from novice clinicians. Future studies should examine child outcomes in under-resourced community settings with larger samples and explore the extent to which inner- and outer-setting factors (e.g., federal policy, agency implementation climate) affect implementation in these settings.

### 4.2. Caregiver and Family Outcomes

NDBIs involve a combination of directive (i.e., behavioral) and responsive (i.e., developmental) strategies (Schreibman et al., 2015). Directive NDBI strategies, such as expectant waiting and prompting, are associated with significantly greater improvements in child language outcomes (e.g., spontaneous communication acts) than responsive strategies, such as following the child’s lead (Jones et al., 2023). However, caregivers that are taught to use responsive NDBI strategies do so at higher fidelity than caregivers who are taught to implement directive NDBI strategies (Roberts et al., 2023). Thus responsive strategies may be more easily learned by caregivers.

In the present study, caregivers mentioned both types of strategies as being effective at improving child social communication. Caregivers reported that Project ImPACT increased caregiver responsivity and engagement with the child’s interests. They also noted that they felt that the NDBI strategies of following the child’s lead and expectant waiting particularly helped to increase their child’s social engagement and social communication skills. It is interesting to note that only one caregiver in our sample explicitly mentioned prompting (a more complex directive strategy) as a useful strategy, but four of the six caregivers mentioned expectant waiting as a useful strategy. Expectant waiting is a less complex directive strategy that may be more easily learned. This may be an important NDBI strategy to focus on when coaching caregivers, as it is relatively simple to execute, and caregivers note its positive effect on child social communication.

Caregivers reported that the strategy of “following the child’s lead” appeared to increase child social communication skills. Responsive NDBI strategies like following the child’s lead are effective at improving social engagement (e.g., child-initiated joint engagement; Patterson et al., 2014) and are also considered by autistic adults to be a more socially valid and affirming approach (Schuck et al., 2024). Following the child’s lead also aligns with many of the tenets of the neurodiversity movement, which advocates that neurodevelopmental disabilities such as autism should be respected as part of the diversity of human beings (Griffin & Pollak, 2009). Contemporary behavioral interventions have faced criticism from the autistic community due to their use of direct prompting strategies to eliminate and change behaviors to fit societal norms, rather than allowing the child to learn through natural opportunities (Chapman & Bovell, 2022). NDBIs such as Project ImPACT have the potential to bridge the gap between current intervention practices and the neurodiversity movement, by using both behavioral and developmental strategies (Schuck et al., 2021). For example, one caregiver in our sample noted how Project ImPACT taught them how to “come into their [child’s] world.” Results suggest that following the child’s lead may be especially useful in supporting enhanced caregiver–child relationships, in addition to improving child social communication outcomes. Additionally, autistic adults report that responsive strategies such as following your child’s lead are more socially valid and can address some ethical concerns such as obtaining child assent during intervention sessions (Schuck et al., 2024). NDBIs have the potential to address concerns about overly directive strategies in ABA by promoting child choice and shared control between child and adult, in contrast to traditional behavioral models that often place little emphasis on child interest (Schuck et al., 2024).

Some caregivers also taught other family members how to use the NDBI strategies. Teaching NDBI strategies to additional family members may be especially important for families from minoritized and low-income backgrounds who are more likely to live in multigenerational households (Cross, 2018). Moreover, if additional family members learn the NDBI strategies, this will likely lead to increased generalization of skills and improvements in family functioning (e.g., more positive interactions within the family structure). Future work on NDBI implementation in under-resourced communities might benefit from an explicit focus on integrating additional caregivers in the learning process.

### 4.3. Future Research

This study focused on the effectiveness of telehealth-delivered Project ImPACT for improving child outcomes within the Medicaid Autism Benefit. It is important to note the exploratory nature of this pilot study; future research should examine results in a larger, fully powered trial, including exploration of the alignment of NDBIs with neurodiversity-affirming practices and focusing on improving clinician fidelity. Replication with larger samples by independent research teams would enhance the credibility of our results and improve the evidence base of research on the effectiveness of Project ImPACT in under-resourced settings.

Moreover, additional research is needed to understand the importance and acceptability of the goals, procedures, and outcomes (i.e., social validity) of this approach from the perspective of different constituent groups (i.e., caregivers, providers, autistic people) (Wolf, 1978). To our knowledge, only two studies have explicitly explored the social validity of NDBIs from the perspective of autistic people (Schuck et al., 2024; D’Agostino et al., 2025). While both demonstrated fairly positive perspectives about the social validity of NDBIs in general, autistic adults have also noted concerns (e.g., feeling it is inappropriate to withhold reinforcement for acquisition tasks that push for a more complex skill; Schuck et al., 2024). Thus, future research should attend to the social validity of this intervention approach, with a specific focus on neurodiversity, cultural fit, caregiver burden, and potential unintended effects of the intervention, recognizing that perspectives may vary based on the stakeholder’s role, underlying philosophy about autism, and cultural background.

### 4.4. Limitations

This study has several limitations. Although our clinician demographics are typical of the behavioral health workforce in this midwestern state, it is important to acknowledge that the lack of racial and ethnic diversity in clinicians could influence the quality of services and/or family decisions to obtain services, particularly when working with families from underrepresented backgrounds.

We also had a small sample (N = 17 for quantitative data, N = 6 for qualitative data), which likely reduced our power to detect statistically significant effects. This may have also negatively impacted our qualitative analyses (e.g., we may have missed additional themes that could have been brought up if more participants agreed to participate in follow-up interviews). While qualitative experts suggest that 6–10 interviews are sufficient for a small-scale project (Braun & Clarke, 2013), conducting additional interviews would likely have provided greater insight and strengthened our analysis.

This project also took place during the COVID-19 pandemic, which resulted in increased caregiver and family stress and the necessity of using telehealth to deliver the intervention. While Project ImPACT has been effectively implemented via telehealth (Ingersoll et al., 2024), this project represents the first study to our knowledge that examined child and family outcomes of a CM-NDBI in a low-income population when all services were provided via telehealth.

Given our single-case research design, the study also did not control for maturation (i.e., did not include a control group); instead, all child outcomes on the Autism Impact Measure were estimated by comparing scores in the intervention period to the average baseline scores for each domain. Future work should utilize larger samples and a control group to more rigorously evaluate child outcomes in an under-resourced setting like this.

Due to resource constraints, this study also did not utilize masked assessors to evaluate child outcomes, and instead relied on caregiver report on questionnaires and in interviews. Relying solely on caregiver report increases the risk of social desirability bias and could result in caregivers over- or under-representing changes in child skills. Future work should incorporate more rigorous assessments of child skills.

Finally, this sample only included children and caregivers who received services within one particular service system, so the results from this study may not generalize to other systems with different implementation contexts.

## Figures and Tables

**Table 1:** Family demographics.

**Demographics**	**Full Sample** **(N = 21)**		**Qualitative Sample** **(n = 6)**	
	**n**	**%**	**n**	**%**
Caregiver Gender				
Woman	17	81%	6	100%
Man	2	10%	0	0%
Unknown	2	10%	0	0%
Caregiver Age	35.7 yrs (range: 23–54 yrs)			
Caregiver Race				
White	15	71%	4	67%
American Indian or Alaska native	1	5%	0	0%
African American	4	19%	2	33%
Asian	0	0%	0	0%
Native Hawaiian or Other Pacific Islander	0	0%	0	0%
Unknown	2	10%	0	0%
Caregiver Ethnicity				
Not Hispanic or Latinx	19	90%	6	100%
Hispanic or Latinx	0	0%	0	0%
Unknown	2	10%	0	0%
Child Gender				
Girl	7	33%	2	33%
Boy	12	57%	4	67%
Unknown	2	10%	0	0%
Child Age	55 mos (range: 24–76 mos)			
Child Race				
White	15	71%	4	67%
American Indian or Alaska Native	1	5%	0	0%
Black or African American	4	19%	2	33%
Asian	1	5%	0	0%
Native Hawaiian or Other Pacific Islander	0	0%	0	0%
Unknown	2	10%	0	0%
Child Ethnicity				
Not Hispanic or Latinx	19	90%	6	100%
Hispanic or Latinx	2	10%	0	0%
Unknown	2	10%	0	0%
Caregiver’s Education				
Some High School	0	0%	0	0%
High School Graduate	5	24%	1	17%
Some college/specialized training	8	38%	4	67%
4-year college graduate	4	19%	1	17%
Graduate degree	2	10%	0	0%
Unknown	2	10%	0	0%

**Table 2:** Multilevel model predicting child peer interaction challenges over time.

Fixed Effects	*b*	SE	*t*	df	*p*
Intercept	22.53	1.37	16.50	20.28	<0.001
Time (weeks)	−0.25	0.10	−2.39	46.43	0.021
Child Age (months)	−0.18	0.11	−1.62	22.04	0.119
Time × Child Age	0.01	0.01	1.18	45.63	0.244
**Random Effects**	**Variance Component**	**SD**			
Intercept	23.15	4.81			

Note: Higher scores indicate more challenges.

**Table 3:** Multilevel model predicting child communication challenges over time.

Fixed Effects	*b*	SE	*t*	df	*p*
Intercept	40.09	2.70	14.84	17.82	<0.001
Time (weeks)	−0.27	0.15	−1.72	45.04	0.092
Child Age (months)	−0.21	0.21	−0.99	18.68	0.335
Time × Child Age	−0.00	0.01	−0.21	44.55	0.839
**Random Effects**	**Variance Component**	**SD**			
Intercept	105.03	10.25			

Note: Higher scores indicate more challenges.

**Table 4:** Multilevel model predicting child social reciprocity challenges over time.

Fixed Effects	*b*	SE	*t*	df	*p*
Intercept	27.30	1.41	19.36	21.59	<0.001
Time (weeks)	−0.14	0.12	−1.13	46.99	0.265
Child Age (months)	−0.09	0.11	−0.80	24.04	0.431
Time × Child Age	0.01	0.01	1.03	45.98	0.309
**Random Effects**	**Variance Component**	**SD**			
Intercept	22.12	4.70			

Note: Higher scores indicate more challenges.

**Table 5:** Joint display for child outcomes and caregiver perspectives.

Quantitative Data (n = 17)	Intercept	*b* (SE)	Statistics
AIM Communication Challenges	40.09	−0.27 (0.15)	*t*(45.04) = −1.72, *p* = 0.09
AIM Peer Interaction Challenges	22.53	−0.25 (0.10)	*t*(46.43) = −2.39, *p* = 0.02
AIM Social Reciprocity Challenges	27.30	−0.14 (0.12)	*t*(46.99) = −1.13, *p* = 0.27
**Qualitative Themes and Subthemes**	**Theme Count and % of Caregivers (N = 6)**	**Representative Quotes**	**Interpretation**
Project ImPACT changes how caregivers interact with their child	30 (100%)	“The following the lead of him made a world of difference. I was never one to really play with kids and having Project ImPACT now I’m like ‘okay we’re playing Mr. Potato Head, and this is how we do it’… You don’t have to follow the standard rules of the game.”	Caregivers often reported a change in the way they spoke to, played with, and interacted with their child following participation in Project ImPACT. This change in caregiver responsivity likely affected child social communication outcomes, such as increased social engagement and communication. NDBI strategies, such as allowing the child to take the lead of the play, result in a more enjoyable interaction for the child, therefore leading to positive benefits such as increasing interaction time and expanding communication. Caregivers feel that Project ImPACT techniques are relatively easy to teach to other family members, supporting increased generalization of skills.
Project ImPACT teaches caregivers to use NDBI strategies that expand the child’s communication	13 (83%)	“I remember one of the tasks where you just pause for a second, don’t try to help her, just let her get her own words out and she will do that or sign ‘more, please’—things like that, not just handing it to her, but making her use her actual words.”	
The NDBI strategy of following your child’s lead supported child social engagement	6 (67%)	“I’m focusing on her, you know doing things she wants to do, and then the playtime can last a bit longer.”	
Project ImPACT strategies increased children’s social engagement	17 (83%)	“He could always grab my hand and take me to do something. But then, once I get that item down for him, he used to just want to take off with it, but now he’ll be like, you know, ‘Do you want to do this with me?’ type of behavior, and he’ll sit down with the puzzle next to me, instead of running off with it to do his own thing.”	
Project ImPACT increased peer interaction	8 (83%)	“At school, he’s been playing more with other kids.”	
Project ImPACT supported the inclusion of additional family members in the child’s intervention services	5 (50%)	“And not only do I do it, but I encourage others to do it, you know, like my mom, his sister, his brother.”	

## Data Availability

The de-identified data presented in this study are available on request from the corresponding author due to privacy concerns.
